# Clinical Response and Changes of Cytokines and Zonulin Levels in Patients with Diarrhoea-Predominant Irritable Bowel Syndrome Treated with *Bifidobacterium Longum* ES1 for 8 or 12 Weeks: A Preliminary Report

**DOI:** 10.3390/jcm9082353

**Published:** 2020-07-23

**Authors:** Gian Paolo Caviglia, Alessandra Tucci, Rinaldo Pellicano, Sharmila Fagoonee, Chiara Rosso, Maria Lorena Abate, Antonella Olivero, Angelo Armandi, Ester Vanni, Giorgio Maria Saracco, Elisabetta Bugianesi, Marco Astegiano, Davide Giuseppe Ribaldone

**Affiliations:** 1 Department of Medical Sciences, University of Turin, 10124 Turin, Italy; chiara.rosso@unito.it (C.R.); marialorena.abate@unito.it (M.L.A.); antonella.olivero@unito.it (A.O.); giorgiomaria.saracco@unito.it (G.M.S.); elisabetta.bugianesi@unito.it (E.B.); 2 Unit of Gastroenterology, Città della Salute e della Scienza di Torino—Molinette Hospital, 10126 Turin, Italy; alessandra.tucci@outlook.it (A.T.); rinaldo_pellican@hotmail.com (R.P.); armandiangelo91@gmail.com (A.A.); evanni@cittadellasalute.to.it (E.V.); marcoastegiano58@gmail.com (M.A.); 3 Institute of Biostructure and Bioimaging, CNR c/o Molecular Biotechnology Centre, 10126 Turin, Italy; sharmila.fagoonee@unito.it

**Keywords:** IBS, IL-6, IL-8, IL-12p70, intestinal permeability, zonulin

## Abstract

*Bifidobacterium longum* (*B. longum*) ES1 is a probiotic strain capable of modulating microbiome composition, anti-inflammatory activity and intestinal barrier function. We investigated the use of *B. Longum* ES1 in the treatment of patients with diarrhoea-predominant irritable bowel syndrome (IBS-D). Sixteen patients were treated for 8 or 12 weeks with *B. Longum* ES1 (1 × 10^9^ CFU/day). Serum zonulin and cytokines were measured at baseline (T0) and at the end of therapy (T1). Clinical response to therapy was assessed by IBS Severity Scoring System. Interleukin (IL)-6, IL-8, IL-12p70 and tumor necrosis factor (TNF) α levels decreased from T0 to T1, irrespective of treatment duration (*p* < 0.05), while zonulin levels diminished only in patients treated for 12 weeks (*p* = 0.036). Clinical response was observed in 5/16 patients (31%): 4/8 (50%) treated for 12 weeks and 1/8 (13%) treated for 8 weeks. Abdominal pain improved only in patients treated for 12 weeks (5/8 vs. 0/8, *p* = 0.025), while stool consistency improved regardless of therapy duration (*p* < 0.001). In conclusion, the results of this pilot study showed, in IBS-D patients treated for 12 weeks with *B. longum* ES1, a reduction in the levels of pro-inflammatory cytokines, and intestinal permeability as well as an improvement in gastrointestinal symptoms, but further studies including a placebo-control group are necessary to prove a causal link.

## 1. Introduction

Irritable bowel syndrome (IBS) is a functional gastrointestinal (GI) disorder affecting approximately 15–25% of the population, with higher prevalence in the female gender [1]. The pathophysiological mechanisms underlying IBS are not completely known. Different factors may participate in disease onset and perpetuation, including genetics, intestinal microbiota and low-grade inflammation [2]. In addition, the role of the intestinal barrier in the pathogenesis of IBS is being increasingly recognised [3]. The loss of barrier integrity allows an increased passage of luminal antigens into the intestinal mucosa, hence stimulating the immune response and sensitizing the afferent nerve fibres [4]. 

Zonulin is a 47-kDa protein involved in the regulation of tight junctions (TJs), the primary determinants of paracellular permeability [5]. Serum zonulin is increased in various diseases in which alteration of intestinal permeability is central, including coeliac disease (CD), inflammatory bowel diseases (IBDs) and type 1 diabetes mellitus [6,7,8]. Moreover, higher serum zonulin levels were seen in patients with IBS compared to controls. Importantly, zonulin levels were directly correlated with the severity of bowel habits in patients with diarrhoea-predominant irritable bowel syndrome (IBS-D) [9].

Many studies have reported the presence of moderate immuno-inflammatory activity in both large and small intestine of patients with IBS [10]. Accordingly, patients with IBS showed increased levels of pro-inflammatory cytokines including interleukin (IL)-1b, tumour necrosis factor-alpha (TNFα), IL-6 and IL-8, and reduced levels of anti-inflammatory cytokines such as IL-10 [11], IL-5 and IL-13 [10]. Through the release of inflammatory cytokines such as TNFα and interferon-gamma (IFNγ), T lymphocytes contribute to TJ dysfunction [12,13].

Emerging literature data support a pathophysiological role of microbiome in IBS. The dysbiosis observed in subjects with IBS is characterized by reduced biodiversity, an increase in Bacteroides and Clostridia, and a reduction in Bifidobacteria [14,15]. There is also evidence of the ability of microbiome to regulate the intestinal barrier. The alteration of the commensal microbiota entails the presence of a morphologically aberrant intestinal mucosa, characterized by shorter ileal villi and smaller intestinal crypts, as demonstrated in germ-free mice [16]. In vitro studies showed that eubiotic microbiome favoured cell renewal process and expression of junctional proteins and mucins [17]. The reduced concentration in Bifidobacteria was associated with the severity of abdominal pain and number of bowel movements. Thus, targeting Bifidobacteria in the intestinal tract may alleviate microbiota-related diseases [18].

The rationale for the use of *Bifidobacterium longum* (*B. longum*) ES1 [19] in patients with IBS-D lies in its ability to modulate the microbiome improving intestinal dysbiosis [20], exert anti-inflammatory activity through the down-regulation of TNF-alpha and the up-regulation of IL-10 [21], and restore the integrity of the intestinal barrier, inducing the synthesis of TJ proteins [22,23].

Several meta-analyses have reported on the effectiveness and safety of probiotics in patients with IBS, especially for products containing Bifidobacteria and Lactobacilli. However, there was significant inter-study heterogeneity, warranting cautious interpretation of the findings. The heterogeneity mainly regarded differences in subgroup analyses of probiotics type, combination and dose, IBS subtype, symptomatic assessment scores and treatment duration [24,25]. Finally, not only probiotics but also prebiotics, such as inositol and beta-glucan, have been shown to improve symptoms in patients with IBS and in patients with concurrent IBD and IBS [26,27].

The aim of this pilot study was (1) to assess the use of *B. longum ES1* treatment in a homogeneous group of patients with IBS-D, by evaluating clinical response, through the use of validated questionnaires for IBS, and serological response, by the determination of serum levels of inflammatory cytokines and zonulin, in order to analyse potential effects on immune modulation and restoration of the intestinal barrier, respectively, and (2) to evaluate differences in response depending on the treatment duration. The results of this pilot study will allow us to define the optimal duration of probiotic therapy and to calculate the necessary sample size to conduct a subsequent randomized, double-blind, placebo-controlled clinical trial.

## 2. Materials and Methods

### 2.1. Patients

We carried out a prospective study at the Gastroenterology Unit of “A.O.U. Città della Salute e della Scienza di Torino” hospital, Italy from April 2019 to October 2019.

Patients affected by IBS-D were recruited and treated with a strain-specific probiotic therapy for 8 or 12 weeks. Probiotic therapy consisted of daily administration of 1 × 10^9^ colony-forming unit (CFU) of *B. longum* ES1 away from meals. Inclusion criteria were: age between 16 and 65 years old, diagnosis of IBS-D according to the Rome IV criteria and Bristol stool scale [28], body mass index (BMI) < 30 kg/m^2^, willingness to sign the informed consent to participate to the study. Exclusion criteria were: history of GI surgery, diagnosis of IBD or CD, thyroid diseases, diverticular disease, small intestine bacterial overgrowth (SIBO), colorectal cancer, other clinically relevant diseases, treatment with drugs that alter intestinal function (e.g., opiates, anticholinergics and laxatives), treatment with antibiotics (any previous antibiotic therapy should have been discontinued at least 4 weeks before the start of probiotic therapy) and other pre/probiotics (other pre/probiotics therapy should have been discontinued at least 2 weeks before starting therapy), pregnancy/breastfeeding.

Clinical history, data on physical examination, recent biochemical examinations and signed informed consent were collected. Disease severity was assessed at baseline with the Functional Bowel Disorder Severity Index (FBDSI) questionnaire [29]. At baseline (T0) and after 8 or 12 weeks of probiotic therapy (T1), patients were evaluated with Irritable Bowel Syndrome Severity Scoring System (IBS-SSS) questionnaire for the clinical evaluation of disease [30] and Irritable Bowel Syndrome Quality of Life (IBS-QoL) questionnaire for life quality assessment [31]. IBS-QoL consists of 34 items and 8 domains; for the purpose of analysis, we considered the overall questionnaire score. The final raw scores of IBS-QoL were presented on a scale between 0 (poor quality of life) and 100 (maximum quality of life).

All patients underwent baseline venous sampling (T0) and after 8 or 12 weeks of therapy (T1); serum was collected in polypropylene 2 mL tubes labelled with the study participant identification code and stored at −80 °C until analysis.

The study followed the principles of the Declaration of Helsinki and was approved by the local ethics committee (Comitato Etico Interaziendale A.O.U. Città della Salute e della Scienza di Torino—A.O. Ordine Mauriziano—A.S.L. Città di Torino) (approval code 0056924).

### 2.2. Measurement of Serum Zonulin and Cytokines

Serum zonulin was assessed by competitive enzyme-linked immunosorbent assay (ELISA) (IDK^®^ Zonulin ELISA Kit, Immunodiagnostik AG, Bensheim, Germany) according to the manufacturer’s instructions. Concentrations were calculated using a four-parameter algorithm, and the results were given in ng/mL, as previously reported [8]. The cytokine panel, including IL-6, IL-8, IL-10, IL-12p70, IL-23, IL-33, IFNγ and TNFα, was measured in serum samples by Bio-Plex^®^ Multiplex Immunoassay (Bio-rad Laboratories, Hercules, CA, USA) on a Luminex^®^ 200 system (Luminex Corporation, Austin, TX, USA). Individual standard curves were generated for each cytokine; the results are given in pg/mL [32]. Personnel performing laboratory investigations were blind to all the characteristics of the patients included in the study.

### 2.3. Outcomes

Clinical and serological outcomes were considered in the study. The clinical outcomes included an IBS-SSS score decrease of ≥50 points, reduction of at least 30% in abdominal pain [33] and bloating by a visual analogic scale (VAS), normalization of stool shape by Bristol Stool Scale and IBS-QoL overall score increase ≥14 points [34,35]. The serological outcomes included the variation in inflammatory cytokines and zonulin levels during the follow-up period to assess potential changing of immune-inflammatory activity and of intestinal permeability, respectively. Both clinical and serological outcomes were evaluated in the overall study population and according to treatment duration.

### 2.4. Statistical Analysis

Continuous variables were expressed as median and 95% confidence interval (CI) or as mean ± standard deviation (SD). The normality of data distribution was tested by D’Agostino-Pearson test. The comparison between quantitative variables was carried out using the Student *t*-test; for the comparison between values at T0 and at T1, the Student *t*-test for paired measurements was used. The comparison between qualitative variables was carried out by Fisher exact test or chi-squared test for trend where appropriate. Nonparametric variables were analysed with the Wilcoxon test for paired samples and with the Mann-Whitney test for independent samples. The correlation between quantitative variables was carried out by non-parametric Spearman test. The results of all analyses were considered significant for *p* values < 0.05. All statistical analyses were performed using MedCalc software version 18.9.1 (MedCalc Software bvba, Ostend, Belgium; http://www.medcalc.org; 2018).

## 3. Results

Sixteen patients aged between 16 and 59 years were enrolled. Basal characteristics of the included cohort are reported in Table 1. Most patients were female (*n* = 10/16, 38%). Patients mainly had normal weight (BMI < 25 kg/m^2^) and all of them consumed a Mediterranean diet [36]. No dietary changes were made prior to study initiation. According to FBDSI score, severity of IBS was mild in 1 patient (6%), moderate in 8 patients (50%), and severe in 7 patients (44%). Anamnestic collection showed that 4 of the 16 enrolled patients (25%) had lactose intolerance confirmed by hydrogen breath test (HBT). All affected patients had reported little or no benefit after the elimination of lactose from diet in the previous years, and no changes in the diet were performed during the study period.

**Figure 1:**
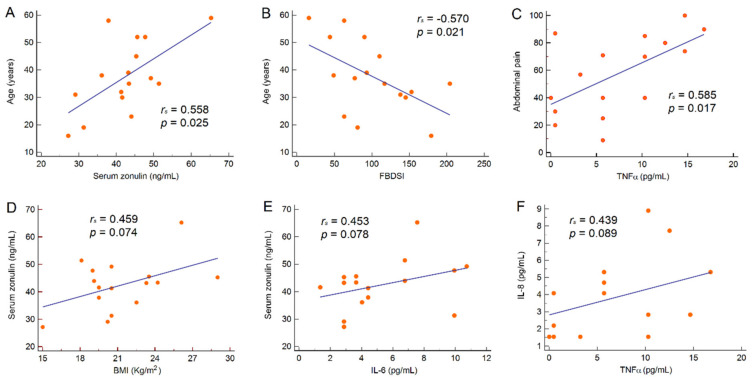
Correlation between age and serum zonulin levels (**A**), age and FBDSI (**B**), abdominal pain intensity and TNFα values (**C**), serum zonulin levels and BMI (**D**), serum zonulin and IL-6 values (**E**) and IL-8 and TNFα values (**F**). Abbreviations: Body Mass Index (BMI), Functional Bowel Disorder Severity Index (FBDSI), interleukin (IL), tumour necrosis factor-alpha (TNFα).

As expected, we observed a positive correlation between FBDSI and IBS-SSS (*r_s_* = 0.658, 95% CI 0.241–0.870, *p* = 0.006) and an inverse correlation between IBS-SSS and overall IBS-QoL score (*r_s_* = −0.550, 95% CI −0.822–−0.074, *p* = 0.027). Age positively correlated with serum zonulin levels (*r_s_* = 0.558, 95% CI 0.086–0.825, *p* = 0.025) and negatively correlated with FBDSI (*r_s_* = −0.570, 95% CI −0.831–−0.104, *p* = 0.021). A significant positive correlation was also observed between abdominal pain intensity and TNFα levels (*r_s_* = 0.585, 95% CI 0.126–0.838, *p* = 0.017). Only a slight positive trend was observed regarding the correlation between serum zonulin and BMI (*r_s_* = 0.459, 95% CI −0.047–0.778, *p* = 0.074), between serum zonulin and IL-6 values (*r_s_* = 0.453, 95% CI −0.055–0.775, *p* = 0.078) and between IL-8 and TNFα values (*r_s_* = 0.439, 95% CI −0.072–0.768, *p* = 0.089). No other significant correlations were observed between serum zonulin or cytokines levels and other demographic and clinical characteristics of the study population (Figure 1).

At baseline, no significant difference was observed between patients undergoing 8 weeks and 12 weeks of treatment regarding severity of disease, serum zonulin levels and cytokines values (Table 2).

The changes of clinical parameters, serum zonulin and cytokines levels from baseline to end of therapy in the overall population of patients with IBS-D treated with *B. longum* ES1 are reported in Table 3.

The clinical response to probiotic therapy, in terms of IBS-SSS score decrease of ≥50 points, was observed in 5/16 patients (31%), of whom 4/8 (50%) were treated for 12 weeks and 1/8 (13%) was treated for 8 weeks (*p* = 0.282); indeed, a trend towards IBS-SSS score reduction from T0 to T1 was found only in patients treated for 12 weeks (236 ± 67 vs. 189 ± 80, *p* = 0.072). An improvement ≥30% of the abdominal pain intensity was found in 5/16 (31%) patients, all of them treated for 12 weeks (5/8; 63%); consistently, patients treated for 12 weeks showed a significant reduction in the intensity of abdominal pain compared to those treated for 8 weeks (from 63 ± 28 to 34 ± 28, *p* = 0.020 and from 52 ± 30 to 62 ± 24, *p* = 0.132, respectively). Conversely, no significant improvement in bloating was reported (*p* = 0.340), independent of therapy duration. An overall IBS-QoL score increase of ≥14 points was obtained only in 1/16 (6%), who was treated for 12 weeks. Stool consistency improved regardless of the duration of therapy (*p* < 0.001). Nine out of 16 (56%) patients normalized stool consistency (type 3 or 4 according to Bristol Stool Scale): 5/8 (63%) were treated for 12 weeks, whereas 4/8 (50%) were treated for 8 weeks (*p* = 0.626). None of the patients reported adverse events during or after treatment.

**Figure 2:**
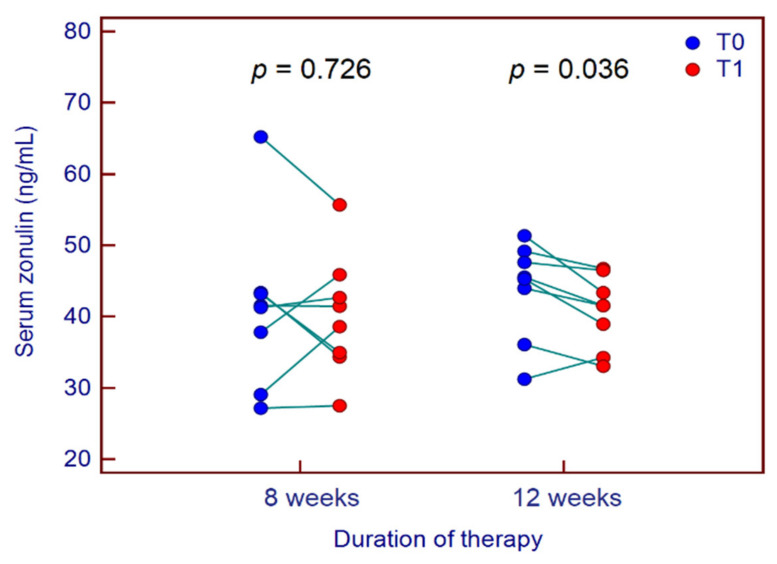
Variation of serum zonulin levels from baseline (T0) to end of therapy (T1) in patients with IBS-D treated with *B. longum* ES1 for 8 weeks or 12 weeks.

Finally, we observed a significant decrease in IL-6, IL-8, IL-12 and TNFα values from baseline to end of therapy, irrespective of treatment duration. Conversely, no significant reduction was found in serum zonulin levels in the overall study population; however, in patients treated for 12 weeks, serum zonulin decreased significantly from 43.8 ± 6.8 ng/mL at T0 to 40.8 ± 5.0 ng/mL at T1 (*p* = 0.036) (Figure 2).

## 4. Discussion

In this pilot study, we found an improvement in overall symptoms of IBS and an improvement in the immune-inflammatory state and integrity of intestinal barrier. These aspects were especially significant in patients treated with *B. longum* ES1 for 12 weeks versus 8 weeks.

Accumulating evidence suggests that commensal bacteria may play a role in IBS and that specific probiotic therapy is able to improve GI symptoms in such patients [37]. Indeed, a recent systematic review showed that patients with IBS had lower microbial α-diversity in both stool and intestinal mucosal samples, with an overall microbial profile characterized by increased levels of Firmicutes and decreased levels of Bacteroidetes compared to healthy subjects [38]. On the other hand, there is a lack of consistency among the gut microbiota fingerprints reported in different studies, probably due to different study designs, different methods used for bacterial profiling (including samples storage, DNA extraction and sequencing) and different statistical approaches [39]. In 48% of patients with IBS-D, it has been shown that probiotic therapy provided adequate relief of overall IBS symptoms, amelioration of stool consistency and a trend towards improved quality of life [40]. More recently, Giannetti et al. reported that the administration of a probiotic mixture of *Bifidobacterium infantis* M-63, *breve* M-16V, and *longum* BB536 in patients with IBS decreased abdominal pain frequency and improved QoL in a significantly higher proportion of patients, when compared with placebo [41]. Moreover, Pinto-Sanchez et al. showed that probiotic therapy (*B. longum* NCC3001) improved psychiatric comorbidities and increased quality of life in patients with IBS [42]. Consistently, we observed an improvement in IBS symptoms especially after 12 weeks of therapy with *B. longum* ES1. However, compared to previous studies, we did not observe an amelioration in the quality of life of our population. Likely, the probiotic mixture or specific strain beneficial for each patient remains to be determined.

Human and animal studies support the concept that a low-grade inflammation may perturb GI reflexes and activate the visceral sensory system contributing to the altered GI physiology and hypersensitivity underlying IBS [43,44]. Moreover, a recent study on murine model demonstrated the important role of neuron-glial communication mediated by TNFα and glial activation in visceral inflammatory hypersensitivity [45]. Consistently, we found a significant positive correlation between abdominal pain intensity and TNFα levels. Several studies have reported an imbalance in pro-inflammatory cytokines, such as IL-6, IL-8 and TNFα, in patients with IBS compared to controls [46,47,48,49]. Unfortunately, in the present study, we were not able to substantiate this data due to the lack of a healthy control population. Lastly, we found a positive correlation between IL-8 and TNFα, in accordance with the chemotactic and activation function performed by IL-8 against neutrophils, which in turn produce TNFα. Treatment with *B. longum* ES1 led to a significant reduction in cytokine levels from baseline to the end of therapy, contributing to the clinical improvement observed in our population. Taken together, these results confirm both the low-grade inflammation described in patients with IBS [36] and the anti-inflammatory effect of *B. longum* ES1 and, overall, support probiotic therapy as a valid treatment option in patients with IBS.

Finally, in the intricate puzzle of IBS pathophysiology, it has been shown that altered intestinal permeability plays an essential role, particularly in its diarrhoea-predominant variant. Indeed, several case-control studies showed that intestinal permeability measured by multi-sugar absorption test increased in patients with IBS-D compared to healthy controls [50,51]. Moreover, Linsalata et al. identified two different IBS-D subtypes that showed different inflammatory status according to intestinal permeability function, further supporting the concept that an impaired GI barrier may allow easier passage of luminal antigens that, in turn, may elicit an inflammatory response influencing the course of the disease [52]. In support of this issue, we found a positive correlation between serum zonulin, a marker of intestinal permeability, and IL-6, secreted by macrophages as a result of the Pathogen Associated Molecular Patterns (PAMPs) binding to the Toll-like receptors. Moreover, we observed a positive correlation between age and zonulin levels. A likely explanation for these findings could lie in a prolonged period of exposure to PAMPs. More recently, it has been reported that treatment with *B. longum* BB536 and *Lactobacillus rhamnosus* HN001 with vitamin B6, compared to placebo, significantly improved GI symptoms and restored intestinal permeability in patients with IBS, as deduced by the improved percentage of sucralose recovery [53]. To date, very few studies have investigated serum zonulin concentration in patients with IBS. Singh et al. showed that serum zonulin levels in patients with IBS were higher compared with healthy controls and comparable to those with active coeliac disease [9]. Taking into account the results on serum zonulin concentration from previous studies conducted at our Center on IBD patients, we observed that serum zonulin concentration in patients with IBS-D was comparable to that found in patients with IBD (43.3 (95% CI 37.3–46.4) vs. 45.3 (95% CI 43.5–47.8) ng/mL, respectively) [30] and higher than those found in healthy controls (8.6 (95% CI 7.2–10.5) ng/mL) [8]. In the present study, a significant reduction in serum zonulin concentration was observed in patients who received probiotic therapy for 12 weeks and not in those treated for 8 weeks. These findings suggest the presence of an altered intestinal permeability in IBS-D patients, and we observed an improvement of the barrier integrity in patients treated for 12 weeks.

The main limitations of the current study are the limited sample size and the lack of a placebo-treated control group, to provide definitive conclusions on the real efficacy of *B. longum* ES1 treatment in our population. We observed a significant improvement of inflammatory status and stool consistency, irrespective of the treatment duration. None of the clinical outcomes allow any inference on the effect on IBS natural history in view of duration of therapy, nature of IBS symptoms (waxing and waning), and marginal differences. Since this was a pilot study, its aim was to evaluate whether our hypothesis could be sufficiently supported to justify a detailed investigation. Such data are promising and set the basis for controlled clinical trials with broader case studies.

## 5. Conclusions

In conclusion, our preliminary results showed a reduction in the levels of pro-inflammatory cytokines in the overall study cohort and possibly an improvement in intestinal permeability and gastrointestinal symptoms in a subgroup of patients with IBS-D. Further randomized, double-blind, placebo-controlled studies are fundamental to validating the results of this pilot study on larger groups of patients with IBS-D.

## Figures and Tables

**Table 1:** Baseline characteristics of the included patients.

Characteristics	*n* (%)
Patients, *n*	16
Female gender, *n* (%)	10 (62)
Age (mean years ± SD)	37.6 ± 15.6
BMI (mean kg/m^2^ ± SD)	21.3 ± 3.4
Married/in a relationship, *n* (%)	4 (25)
Caesarean childbirth, *n* (%)	5 (31)
History of breastfeeding, *n* (%)	7 (44)
Current smokers, *n* (%)	0, (0)
Lactose intolerance, *n* (%)	4 (25)
Atopy, *n* (%)	5 (31)
FBDSI (mean ± SD)	101 ± 52
IBS-SSS (mean ± SD)	289 ± 91
Abdominal pain (mean ± SD)	57 ± 28
IBS-QoL overall score (mean ± SD)	71 ± 16
Bristol Stool Scale (mean ± SD)	6 ± 1
Zonulin (ng/mL, mean ± SD)	42.5 ± 9.3
IL-6 (pg/mL, median, 95% CI)	4.22, 2.87–7.06
IL-8 (pg/mL, median, 95% CI)	3.47, 1.96–5.32
IL-12p70 (pg/mL, median, 95% CI)	1.77, 0.57–2.16
TNFα (pg/mL, median, 95% CI)	5.69, 2.23–11.09

Abbreviations: body mass index (BMI), confidence interval (CI), Functional Bowel Disorder Severity Index (FBDSI), interleukin (IL), Irritable Bowel Syndrome Quality of Life (IBS-QoL), Irritable Bowel Syndrome Severity Scoring System (IBS-SSS), *n* (number), standard deviation (SD), tumour necrosis factor-alpha (TNFα).

**Table 2:** Comparison of disease severity, IBS-QoL overall score, Bristol Stool Chart, serum zonulin levels and cytokines values, assessed at baseline (T0), between the two groups of patients treated with *B. Longum* ES1 for 8 or 12 weeks.

Parameters	T0 (8w)	T0 (12w)	*p* Values
Patients (*n*)	8	8	
Disease severity (mild/moderate/severe)	1/2/5	0/6/2	0.248
IBS-QoL overall score (mean ± SD)	65 ± 17	78 ± 13	0.118
Bristol Stool Scale (mean ± SD)	6 ± 1	7 ± 1	0.150
Zonulin (ng/mL, mean ± SD)	41.1 ± 11.60	43.8 ± 6.8	0.570
IL-6 (pg/mL, median, 95% CI)	3.26, 2.58–5.01	6.77, 3.50–10.09	0.060
IL-8 (pg/mL, median, 95% CI)	3.46, 1.55–5.77	3.78, 1.55–5.99	0.670
IL-12p70 (pg/mL, median, 95% CI)	1.37, 0.57–3.68	1.77, 0.57–2.16	0.830
TNFα (pg/mL, median, 95% CI)	5.69, 0.47–10.70	7.99, 0.38–15.06	0.460

Abbreviations: interleukin (IL), confidence interval (CI), number (*n*), non-responder (NR), responder (R), standard deviation (SD), tumour necrosis factor-alpha (TNFα), week (w).

**Table 3:** Changes of questionnaires scores, serum zonulin levels and cytokines values from T0 to T1 in the overall study population.

Parameters	T0	T1	*p* Values
Patients (*n*)	16	16	
IBS-SSS (mean ± SD)	289 ± 91	263 ± 118	0.103
Pain intensity (mean ± SD)	57 ± 28	47 ± 31	0.216
Bloating (mean ± SD)	39 ± 30	44 ± 32	0.340
IBS-QoL overall score (mean ± SD)	71 ± 16	73 ± 17	0.299
Bristol Stool Scale (mean ± SD)	6 ± 1	4 ± 1	<0.001 *
Zonulin (ng/mL, mean ± SD)	42.5 ± 9.3	40.5 ± 6.8	0.179
IL-6 (pg/mL, median, 95% CI)	4.22, 2.87–7.06	0.01, 0.01–3.65	<0.001 *
IL-8 (pg/mL, median, 95% CI)	3.47, 1.96–5.32	0.01, 0.01–1.12	0.011 *
IL-12p70 (pg/mL, median, 95% CI)	1.77, 0.57–2.16	0.01, 0.01–0.21	0.001 *
TNFα (pg/mL, median, 95% CI)	5.69, 2.23–11.09	0.01, 0.01–5.69	0.034 *

Abbreviations: confidence interval (CI), interleukin (IL), Irritable Bowel Syndrome Quality of Life (IBS-QoL), Irritable Bowel Syndrome Severity Scoring System (IBS-SSS), number (*n*), standard deviation (SD), tumour necrosis factor-alpha (TNFα), statistically significant (*).
